# ADT-OH, a hydrogen sulfide-releasing donor, induces apoptosis and inhibits the development of melanoma in vivo by upregulating FADD

**DOI:** 10.1038/s41419-020-2222-9

**Published:** 2020-01-16

**Authors:** Fangfang Cai, Huangru Xu, Nini Cao, Xiangyu Zhang, Jia Liu, Yanyan Lu, Jia Chen, Yunwen Yang, Jian Cheng, Zi-Chun Hua, Hongqin Zhuang

**Affiliations:** 1 The State Key Laboratory of Pharmaceutical Biotechnology, College of Life Sciences, Nanjing University, Nanjing, China; 2 Institute of Neuroscience, Soochow University, Suzhou, China; 3 Changzhou High-Tech Research Institute of Nanjing University and Jiangsu TargetPharma Laboratories Inc., Changzhou, 213164 China

**Keywords:** Cancer therapy, Apoptosis

## Abstract

Hydrogen sulfide (H_2_S) is now widely considered the third endogenous gasotransmitter and plays critical roles in cancer biological processes. In this study, we demonstrate that 5-(4-hydroxyphenyl)-3H-1,2-dithiole-3-thione (ADT-OH), the most widely used moiety for synthesising slow-releasing H_2_S donors, induces melanoma cell death in vitro and in vivo. Consistent with previous reports, ADT-OH inhibited IκBɑ degradation, resulting in reduced NF-κB activation and subsequent downregulation of the NF-κB-targeted anti-apoptotic proteins XIAP and Bcl-2. More importantly, we found that ADT-OH suppressed the ubiquitin-induced degradation of FADD by downregulating the expression of MKRN1, an E3 ubiquitin ligase of FADD. In addition, ADT-OH had no significant therapeutic effect on FADD-knockout B16F0 cells or FADD-knockdown A375 cells. Based on these findings, we evaluated the combined effects of ADT-OH treatment and FADD overexpression on melanoma cell death in vivo using a mouse xenograft model. As expected, tumour-specific delivery of FADD through a recombinant *Salmonella* strain, VNP-FADD, combined with low-dose ADT-OH treatment significantly inhibited tumour growth and induced cancer cell apoptosis. Taken together, our data suggest that ADT-OH is a promising cancer therapeutic drug that warrants further investigation into its potential clinical applications.

## Introduction

Melanoma is one of the most prevalent forms of skin cancer, and approximately 232,100 (1.7%) of all newly diagnosed primary malignant cancer cases worldwide are cutaneous melanoma^1^. In recent decades, activating v-Raf murine sarcoma viral oncogene homologue B (BRAF) mutations were found in approximately one-half of all cutaneous melanomas^2,3^. Treatment with selective BRAF inhibitors (such as vemurafenib and dabrafenib) combined with inhibitors of the downstream MAP kinase MEK (such as trametinib and cobimetinib) has significantly improved the treatment response and overall survival of cutaneous melanoma^4–7^. Recently, combinations of immune checkpoint inhibitors, such as anti-PD1 and anti-CTLA4, have shown the ability to significantly prolong overall survival^8^. However, melanoma is still a deadly disease in the metastatic stage because of frequent tumour relapse and therapy resistance, upon which death follows within only a few months and results in approximately 55,500 cancer deaths worldwide^1,9^. Thus, further improvements and particularly new, more effective combination treatments should be developed to improve the overall survival of cutaneous melanoma.

Several different cellular mechanisms may contribute to the therapy resistance of melanoma, and among these, apoptosis deficiency might be considered the major factor^10,11^. Two major pathways are involved in apoptosis: the intrinsic (induced by cellular or DNA damage) and extrinsic (initiated by the interaction of death ligands with death receptors) apoptotic pathways^12^. Deficiency of apoptosis in cancer cells usually results in therapy resistance of melanoma, implying that elimination of cancer cells through pro-apoptotic programmes might be an attractive anticancer strategy. In fact, several chemotherapeutic drugs, including BRAF inhibitors, have been reported to cause cellular or DNA damage, leading to the induction of intrinsic cell pro-apoptotic pathways^13–15^. In addition, our previous study has shown that tumour-targeted delivery of Fas-associated protein with death domain (FADD), which is the key adaptor protein for the apoptotic signal transduction that activates caspase-8 and leads to extrinsic apoptosis, suppresses B16F10 melanoma cells by enhancing auto-initiated apoptosis^16^. However, effective strategies to induce pro-apoptotic programmes of melanoma are still needed.

Over the past few decades, several studies have indicated that hydrogen sulfide (H_2_S) is involved in the regulation of cancer biological processes, including those specific to the progression of human melanoma. Endogenous H_2_S is synthesised by single enzymes, namely, cystathionine β-synthase (CBS) and cystathionine β-lyase, (CSE) and tandem enzymes, cysteine aminotransferase (CAT) and 3-mercaptopyruvate sulfurtransferase (3-MST)^17^. Accumulating evidence indicates that CSE and CBS play important roles in various types of cancer cells. Recently, several studies have also indicated that H_2_S is involved in the regulation of cancer biological processes, particularly in the progression of human melanoma. Moreover, both cancer-promoting and anticancer effects have been described for H_2_S^18–20^. Low concentrations of either endogenous or exogenous H_2_S promote tumour progression, mainly in the following ways: first, promoting angiogenesis by increasing the expression of VEGFR;^21,22^ second, promoting tumour growth by improving mitochondrial function in cancer cells;^23^ third, accelerating cancer cell cycle progression by modulating the phosphorylation of PKB/AKT and the action of extracellular signal-regulated kinase (ERK)^24^ and fourth, protecting against apoptosis in tumour cells by reducing intracellular ROS levels, thereby reducing mitochondrial disruption and DNA damage^25^. However, several studies have shown that H_2_S donors, including DATS, GYY4137 and ATB-346, markedly decrease the expression of anti-apoptotic genes, such as those encoding FLICE-inhibitory protein (c-FLIP) and B cell lymphoma gene-2 (Bcl-2), which can induce pro-apoptotic programmes in several cancer cell types^18,26^. In addition, exogenous H_2_S can increase the activity of anion exchangers and sodium/proton exchangers to increase the production of metabolic acids to cause cancer cell death^27^. Moreover, it inhibits the phosphorylation of STAT3 to induce cell cycle arrest^28^. Due to the potential pro-apoptotic activities of H_2_S donors, we wondered whether they, especially in combination with other agents that can alleviate apoptosis deficiency in cancer cells, could provide an effective strategy in the treatment of melanoma.

Therefore, the aim of this study was to evaluate the effect of the combination of H_2_S donors combined with other pro-apoptotic agents in the treatment of melanoma. In our study, 5-(4-hydroxyphenyl)-3H-1,2-dithiole-3-thione (ADT-OH), one of the most widely used moieties for synthesising slow-releasing organic H_2_S donors, was used as an H_2_S donor. The tumour-targeted delivery of FADD was used as a pro-apoptotic agent-based strategy on our previous study^16^. Intriguingly, ADT-OH markedly decreased the expression of makorin ring finger protein 1 (MKRN1), which is an E3 ubiquitin ligase of FADD, resulting in elevated protein levels of FADD. ADT-OH treatment combined with overexpression of FADD significantly induced apoptosis and suppressed the growth of melanoma cells both in vitro and in vivo, providing a potential effective strategy for melanoma therapy.

## Materials and methods

### Bacterial strain, cell lines and reagents

The pcDNA3.1 (−) vector was obtained from Invitrogen (Invitrogen, USA). MKRN1 and FADD cDNAs were subcloned into pcDNA3.1, pcDNA3.1-HA and pcDNA3.1-Flag. The ubiquitin-HA plasmid was purchased from GeneCopoeia (EX-F0214-M06, GeneCopoeia, Inc., USA). The pQE30-NirB plasmid was maintained in our laboratory. Lipid A modified (msbB−), auxotrophic (purI−) *Salmonella typhimurium* VNP20009 (VNP) was obtained from the ATCC (USA) and cultured in modified Luria–Bertani (LB) medium at 37 °C. The hypoxia-inducible expression of *S. typhimurium* VNP20009 VNP-pN-FADD (VNP-FADD) was maintained as described in the previous article^16^. B16F10 and B16F1 (murine melanoma cells), LLC Lewis (murine lung carcinoma cells), 4T1 (murine breast cancer cells), A375 (human melanoma cells), A549, H446 and H1299 (human lung carcinoma cells), MCF-7 (human breast adenocarcinoma cell line), MDA-MB-231 (human breast cancer cells), HCT-116 (human colorectal carcinoma cells), HepG2 (human hepatocellular carcinoma cells), HaCaT (human immortalised epidermal cells), HK2 (human proximal tubule epithelial cells) and MEF (murine embryonic fibroblasts) cell lines were purchased from American Type Culture Collection (ATCC, USA) or maintained in our laboratory and cultured at 37 °C in 5% CO_2_ in a humidified atmosphere in Dulbecco’s modified Eagle’s medium (DMEM, Gibco, Shanghai, China) with 10% foetal bovine serum (FBS, Gibco, Australia), penicillin (100 IU/ml) and streptomycin (100 μg/ml). Cells were transfected with plasmid DNA using PolyJet™ reagent (SignaGen, USA) as described previously^16^. After adding the plasmid to the B16F10 melanoma cells for 6 h, ADT-OH was added. The cells were harvested for the desired experiment until overexpression for 24 h. 5-(4-hydroxyphenyl)-3H-1,2-dithiole-3-thione (ADT-OH) and NaHS were synthesised by Suzhou University and were solubilized in DMSO.

### H_2_S measurements

The MEFs and B16F10 melanoma cells were seeded in 96-well plates and cultured in medium with 10 mM Cys, 10 μM PLP and ADT-OH. Lead acetate paper (RA, Sigma, St. Louis, MO, USA) was placed on the plate for 2–24 h and further incubated in a 37 °C CO_2_ incubator. To measure the exact amount of H_2_S released, a H_2_S detection kit (R&D Systems, Abnova, USA) was used. The MEFs and B16F10 melanoma cells were treated with ADT-OH in a time series and a concentration gradient, and the cell supernatant was then collected and detected according to the manufacturer’s instructions.

### Detection of oxidative stress

The B16F10 melanoma cells were seeded in 12-well plates and cultured in medium with specific concentrations of ADT-OH for 24 h. Then, the reactive oxygen species (ROS) assay kit (Beyotime, China) was used to detect the accumulation of ROS according to the manufacturer’s instructions.

### Cell proliferation and apoptosis assay

Cell proliferation was measured by CCK-8 assay. Cells were seeded on 96-well plates (5 × 10^3^ cells/well) and treated with ADT-OH (0.8–100 μM) for 24, 48 or 72 h before 10 μl of CCK-8 (Sigma, Milan, Italy) was added. After 1 h of incubation, the cells on the plates were measured using a microplate spectrophotometer (Titertek Multi-skan MCC/340) equipped with a 450 nm filter. Apoptosis was detected after the B16F10 melanoma cells were treated with ADT-OH (0.8, 3.2, 12.5 and 50 μM) for 24 h with enhanced green fluorescent protein-conjugated Annexin V (BD Pharmingen, San Diego, CA, USA) according to the manufacturer’s instructions.

### CRISPR/Cas9 generated FADD-knockout B16F10 cells

FADD-knockout B16F10 and A549 cells were generated as described in previous literature^29^. Briefly, two FADD gRNAs (guide RNAs) and one negative control (NC) gRNA were designed with 5′ and 3′ BsaI restriction site overhangs (Supplementary Table 1), annealed and ligated into a pX601-GFP plasmid (Addgene Plasmid #84040). These plasmids were transfected into B16F10 cells, and the cells with green fluorescent genes were sorted by flow cytometry (BD FACSAria II, BD Pharmingen, San Diego, CA, USA). Western blot analysis using FADD antibody was performed to confirm FADD knockout efficiency.

### SiRNAs and transfection

All synthetic siRNAs and NCs were purchased from Shanghai Gene Pharma Co., Ltd. A375 cells were transiently transfected with FADD siRNAs (human FADD siRNA sequence: 5′-CACAGAGAAGGAGAACGCA-3′) using Lipofectamine 2000 (Invitrogen, USA) according to the manufacturer’s instructions.

### Protein extraction and immunoblotting

Cells or tissues were collected and homogenised with lysis buffer containing 10 mM TRIS-HCl (pH 7.9), 10% glycerol, 0.1 mM EDTA, 100 mM KCl, 0.2% NP-40, 0.5 mM PMSF, 1 mM dithiothreitol (DTT), and 1× protease inhibitor cocktail (Roche, Germany). After 30 min of incubation on ice, whole-cell extracts were pelleted in an Eppendorf microcentrifuge at 12,000 rpm for 10 min at 4 °C, then stored at −20 °C or immediately subjected to sodium dodecyl sulfate polyacrylamide gel electrophoresis. The protein concentration was measured with the bicinchoninic acid method (Pierce, USA). Then, 40 μg of total protein were used for immunoblotting analysis following standard conditions with the following primary antibodies: Bcl-2 (2876, Cell Signalling, USA; dilution 1:1000), Bax (2772, Cell Signalling, USA; dilution 1:1000), Bad (9292, Cell Signalling, USA; dilution 1:1000), FADD (ab124812, Abcam, Cambridge, UK; dilution 1:1000), MKRN1 (SAB2501717, Sigma-Aldrich; dilution 1:1000), IκBα (4812, Cell Signalling, USA; dilution 1:1000), cleaved caspase-8 (8592, Cell Signalling, USA; dilution 1:1000), XIAP (2042, Cell Signalling, USA; dilution 1:1000), cleaved caspase-3 (9662, Cell Signalling, USA; dilution 1:1000), PARP (9542, Cell Signalling, USA; dilution 1:1000); Flag (F3165 mouse; Sigma); ubiquitin (3933, Cell Signalling, USA; dilution 1:1000); β-tubulin (Abgent, Suzhou, China) and β-actin (Abgent, Suzhou, China). The proteins were detected with an ECL Plus Western blot detection system (Tanon, Shanghai, China). The proteins were quantified by measuring the band intensities with ImageJ software.

### Real-time quantitative PCR assay

Total RNA was isolated with TRIzol reagent (Invitrogen), and cDNA was generated using a ReverTra Ace® qPCR RT kit (Toyobo). Real-time quantitative polymerase chain reaction (qPCR) was performed with the primers listed in Supplementary Table 2. Real-time qPCR was performed on a StepOne Real-Time PCR system (Applied Biosystems, USA) with AceQ® qPCR SYBR® Green Master Mix (Vazyme, China). Data were analysed by StepOne 2.1 software (Applied Biosystems, USA) according to the manufacturer’s specifications. β-Actin was used as a control.

### Animal model and treatment

C57BL/6 mice (female, 6–8 weeks of age) were obtained from Model Animal Research Center of Nanjing University (Nanjing, China) and housed under germ-free conditions. Animal care and use were conducted in strict accordance to the ethical guidelines of the Nanjing University Animal Care and Use Committee, and the study protocol was approved by the local institutional review board. The animals were randomly allocated into experimental groups.

Mice were subcutaneously (s.c.) injected in the hind flank region with B16F10 cells (2 × 10^5^ cells/100 μl). Starting on day 1, groups of mice (*n* = 8 per group) were treated orally with equal concentrations and volumes of ADT-OH (37.5 mg/kg, 100 μl per mouse) or vehicle (100 μl per mouse) once every other day. All drugs were suspended in 0.5% carboxymethylcellulose/phosphate-buffered saline (PBS). In addition, when the tumour volume reached approximately 100–150 mm^3^, the mice receiving VNP and VNP-FADD therapy were treated with an intraperitoneal injection of 1 × 10^5^ cfu VNP or VNP-FADD per mouse, according to the following VNP model methods. Tumour volume was measured and a mouse survival curve was generated as described previously^16^.

### VNP model

The VNP and VNP-FADD strains were treated as follows: VNP and VNP-FADD strains in the exponential growth phase were harvested at 1 × 10^8^ cfu, washed twice with PBS, and resuspended in PBS to a concentration of 1 × 10^5^ cfu/100 μl. The mice were subcutaneously (s.c.) injected in the hind flank region with B16F10 cells (2 × 10^5^ cells/100 μl). When the tumour volume reached approximately 100–150 mm^3^, the mice were randomly divided (no fewer than 8 mice per group) and then intraperitoneally injected with 100 μl VNP or VNP-FADD per mouse. An injection of PBS was used in the controls. The combination group also administered ADT-OH at this time. Tumour volume was measured and mouse survival curve was generated as described previously^16^. To investigate the biological distribution of the strains in vivo, the mice were sacrificed 3 d after receiving the inoculation. The liver, spleen and tumour tissues were taken, placed in a homogeniser with sterile PBS, and then diluted with a gradient and applied to Amp plates. Colony counts were performed after the plates had been cultured at 37 °C for 16 h.

### Haematoxylin and eosin (H&E) staining, TUNEL assays and immunofluorescence microscopy

After 15 d of treatment, the tumour-bearing mice were sacrificed, and the tumour tissue was removed. Tumour sections were prepared according to the standard protocol of H&E staining. Terminal deoxynucleotidyl transferase dUTP nick end labeling (TUNEL) assays were performed by using the TUNEL BrightGreen apoptosis detection kit following the manufacturer’s instructions (Vazyme, Nanjing, China). To analyse the level of caspase-3 activation and FADD, tumour sections were stained with anti-cleaved caspase-3 (Cell Signalling Technology, USA) and FADD (Abcam, USA). The second antibody, to cleaved caspase-3 and FADD, was goat anti-rabbit IgG labelled with FITC (Invitrogen, USA).

### Statistical analysis

Experiments were performed at least three times with similar results. The results are presented as the mean ± S.D. or S.E.M. after the analyses were completed with GraphPad Prism 6.0 (Graph Pad Software, San Diego, CA, USA). A log-rank test was used to analyse the effect of treatment on survival time. One-way analysis of variance was used to calculate the significant differences among groups. A value of *p* < 0.05 was considered significant.

## Results

### ADT-OH inhibits melanoma cell proliferation and induces apoptosis

**Fig. 1:**
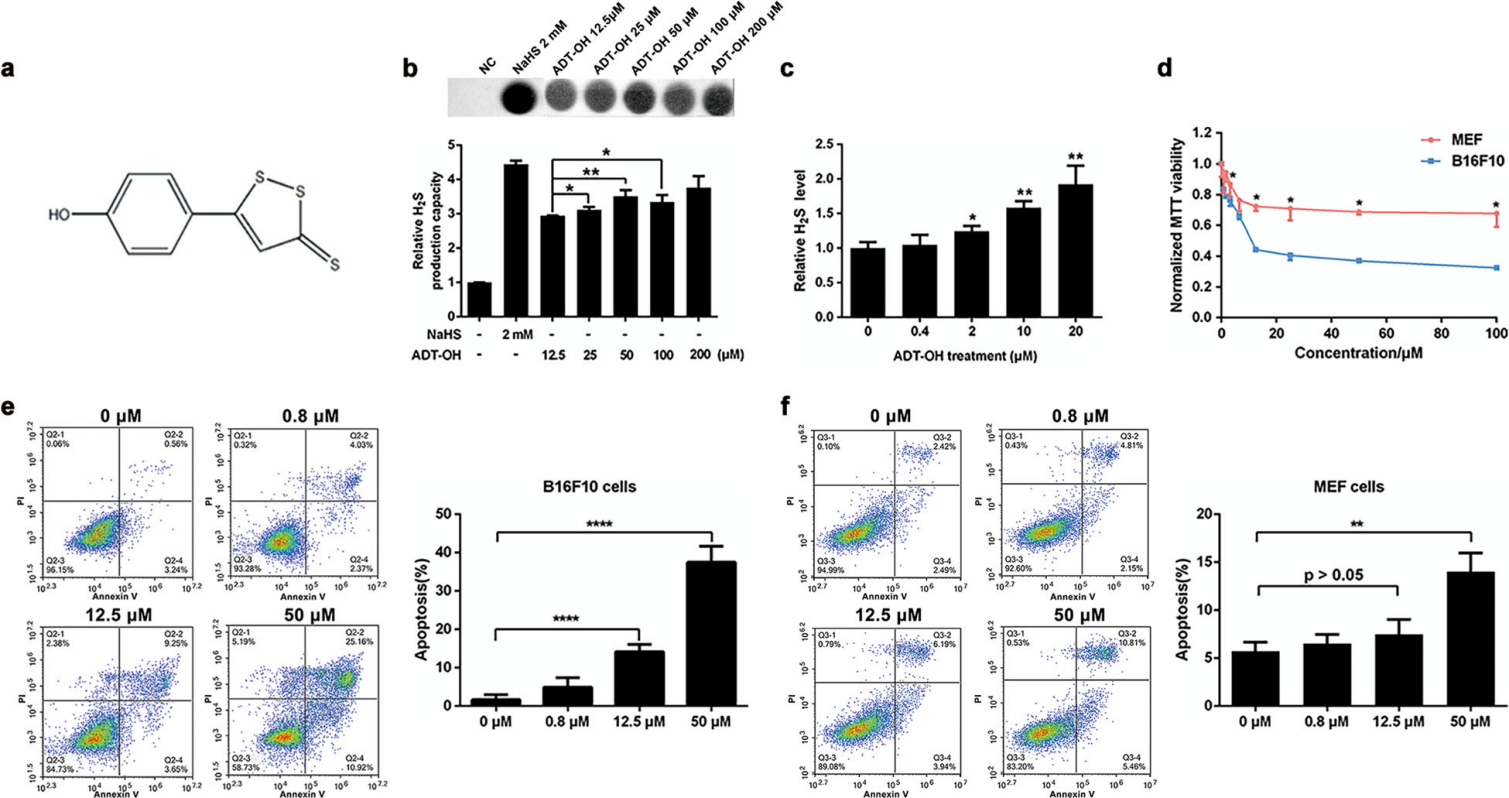
ADT-OH inhibits melanoma cell proliferation and induces apoptosis. **a** The chemical structure of ADT-OH. **b** H_2_S production capacity of ADT-OH acting on MEF cells *via* the lead sulfide method. **c** H_2_S measurement released by B16F10 melanoma cells after ADT-OH treatment in different concentration. **d** MEF and B16F10 melanoma cells were incubated with increasing concentrations of ADT-OH for 24 h. Cell viability was determined by CCK-8 assay. **e** B16F10 melanoma cells were treated with ADT-OH at different concentration (0.8–50 μM) and apoptosis was determined by flow cytometry analysis. And quantitative analysis of apoptosis at various concentrations of exposure to ADT-OH. Experiments (*n* = 3) were performed in triplicate. **f** Representative FACS analysis and quantitative analysis of Annexin V and propidium iodide (PI) staining after ADT-OH treated at different concentration (0.8–50 μM) in MEF cells. Data are represented as mean ± SD for different experiments performed in duplicate. In **b**, *P < 0.05, **P < 0.01 compared with ADT-OH 0.5 μM group; in **c** and **d**, *P < 0.05, **P < 0.01 compared with vehicle group.

The structural component of ADT-OH is shown in Fig. 1a. To detect the release of hydrogen H_2_S sulfide, we used a specific reaction between H_2_S and lead acetate to form a black precipitate (lead sulfide), which can be captured and observed on filter paper containing lead acetate. We found that ADT-OH released H_2_S in a dose-dependent manner (Fig. 1b). In addition, ADT-OH released H_2_S in cells slowly in a concentration- and time-dependent manner (Fig. 1c, Supplementary Fig. 1). To investigate the potential antiproliferative effects of ADT-OH, a CCK-8 assay was performed on various tumour cell lines and a MEF cell line. As shown in Fig. 1d and Supplementary Fig. 2, ADT-OH significantly inhibited the proliferation of a variety of tumour cells, including melanoma cells. In addition, the B16F10 cell line was the most sensitive to ADT-OH treatment and was chosen for further study. Moreover, ADT-OH strongly inhibited the proliferation of the B16F10 cells in a dose-dependent manner but had a slight effect on MEFs (Fig. 1d). Treatment with ADT-OH at a dose of 12.5 μM reduced MEF proliferation by 27.64% in and B16F10 cells by 55.74%, which implies that ADT-OH had a greater inhibitory effect on the proliferation of tumour cells than it did on normal cells. In addition, Fig. 1e shows that ADT-OH induced obvious apoptosis of the B16F10 cells in a dose-dependent manner (15.02% at 12.5 μM and 41.95% at 25 μM). Interestingly, ADT-OH had only a slight effect on the apoptosis of MEFs (Fig. 1f). In addition, the other two normal cell lines, HaCaT and HK2, and the other cancer cell lines, namely, HepG2, MCF-7, HCT-116 and MDA-MB-231, were used to analyse the pro-apoptotic effects of ADT-OH (Supplementary Fig. 3). In a similar pattern to its effect on proliferation, ADT-OH induced tumour cell apoptosis but had a slight effect on the normal cells. These results indicated that tumour cells are more sensitive to ADT-OH, although high concentrations of ADT-OH can induce apoptosis in normal cell lines. Previous studies reported that H_2_S donors increased the production of intracellular ROS; therefore, the production of ROS in the B16F10 cells after ADT-OH treatment was also analysed. As shown in Supplementary Fig. 4, 50 μM ADT-OH increased ROS production slightly, but 12.5 μM ADT-OH did not affect ROS production. The results indicated that there might be other signalling pathways accounting for ADT-OH-induced apoptosis.

### ADT-OH induces extrinsic apoptosis by increasing FADD expression

**Fig. 2:**
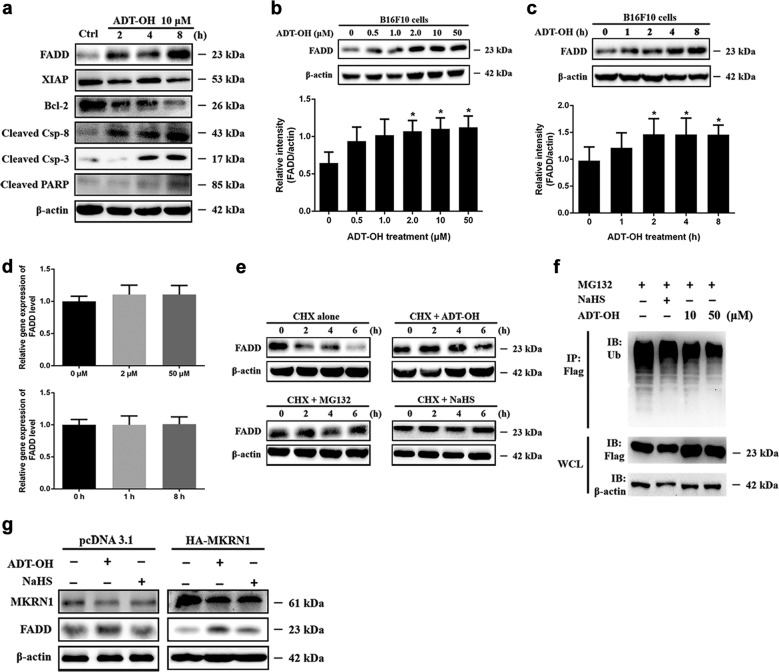
ADT-OH suppresses the ubiquitin degradation of FADD through downregulating MKRN1 expression to trigger extrinsic apoptosis. **a** Western blot analysis of cleaved-caspase 3, cleaved-caspase 8, cleaved-PARP, Bcl-2, XIAP and FADD in B16F10 cells treated with ADT-OH (10 μM) at different time point as indicated. **b**, **c** Western blot analysis of FADD expression in B16F10 cells after ADT-OH treated in dose- (**b** 2 h) and time- (**c** 10 μM) dependent manner. **d** The mRNA levels of FADD in B16F10 cells after ADT-OH treated at different concentrations and time points as indicated. **e** B16F10 cells were treated with 40 μg/ml of CHX for the indicated time to determine protein stability of FADD in the absence or presence of MG132 (10 μM), NaHS (2 mM), and ADT-OH (10 μM). Cells were lysed and analysed by WB using anti-FADD and anti-actin antibodies. **f** B16F10 cells were transfected with the FADD-Flag and HA-Ub plasmid, treated with MG132 (10 μM) for 6 h, NaHS 2 mM or ADT-OH in the the indicated concentrations for 6 h, then lysed in 1% SDS buffer. Flag-FADD was purified by immunoprecipitation using an anti-FLAG antibody, followed by WB analysis using an anti-HA antibody. **g** The effect of ADT-OH on MKRN1 and FADD protein levels by WB analysis. B16F10 cells were transfected with FADD in the absence or presence of MKRN1, NaHS and ADT-OH for 24 h and then treated with CHX (40 μg/ml) for 6 h. *P < 0.05, **P < 0.01. Data are expressed as mean ± SD of three independent experiments.

The pro-apoptotic effect of ADT-OH was further analysed by Western blot analysis. The Western blot analysis results showed that ADT-OH treatment inhibited the activation of NF-κB and the expression of its target genes, namely, XIAP and Bcl-2, in B16F10 cells (Fig. 2a, Supplementary Fig. 5 and Supplementary Fig. 6). These results showed that intrinsic apoptosis was activated by ADT-OH treatment, a finding that was consistent with that of other H_2_S donors reported in previous studies^26,30,31^. In addition, our results showed that the protein levels of cleaved caspase-8 and caspase-3 and poly (adenosine diphosphate-ribose) polymerase (PARP) were all significantly upregulated (Fig. 2a and Supplementary Fig. 5). These results indicated that ADT-OH could induce apoptosis through exogenous stimulation. To explore the mechanisms, the extrinsic apoptosis pathway was analysed, and we found that the protein level of FADD was significantly elevated after ADT-OH treatment (Fig. 2a and Supplementary Fig. 5). Moreover, this effect was time- and dose-dependent (Fig. 2b, c). Similarly, ADT-OH treatment also upregulated the protein levels of FADD in the 4T1, LLC, A549 and HepG2 cell lines (Supplementary Fig. 7). However, the qPCR results showed that the mRNA levels of FADD were not affected by ADT-OH (Fig. 2d).

### ADT-OH inhibits FADD ubiquitin-mediated degradation by regulating MKRN1

To investigate the underlying mechanism by which ADT-OH regulates the protein level of FADD in B16F10 cells, protein stability experiments were performed. We measured the half-life of FADD upon treatment with the protein synthesis inhibitor cycloheximide (CHX). As shown in Fig. 2e, the half-life of FADD was approximately 4 h; however, the degradation of FADD was almost completely blocked by MG132 treatment. Interestingly, ADT-OH and NaHS also inhibited the degradation of FADD (Fig. 2e, Supplementary Fig. 8), suggesting that ADT-OH might be involved in the proteasome degradation pathway of FADD. To investigate the role of ADT-OH on the ubiquitin-mediated degradation of FADD, we performed a FADD ubiquitination-degradation experiment. As shown in Fig. 2f, ADT-OH significantly reduced the ubiquitination-mediated degradation of FADD. FADD ubiquitination and degradation have been reported to be dependent on its E3 ubiquitin ligase, MKRN1^32^. Moreover, we found that increased levels of exogenous MKRN1 significantly induced FADD degradation but that treatment with NaHS or ADT-OH reversed this outcome (Fig. 2g and Supplementary Fig. 9). The mRNA and protein levels of MKRN1 were analysed to determine whether ADT-OH increased the protein level of FADD through MKRN1. As shown in Supplementary Fig. S10, ADT-OH treatment greatly decreased the stability of the MKRN1 protein even though the mRNA level of MKRN1 was decreased by only 15%, indicating that ADT-OH affected the protein level of MKRN1 mainly by decreasing its stability. In summary, ADT-OH reduces the level of ubiquitination of FADD by decreasing the protein stability of MKRN1, which ultimately increases the protein level of FADD. Interestingly, according to the online cancer transcriptome database Oncomine, MKRN1 is overexpressed in stage 4 human uveal melanoma (UVM) but not in skin cutaneous melanoma. In addition, a Kaplan–Meier survival analysis of UVM patients showed that patients with higher expression of MKRN1 exhibited poorer overall survival (Supplementary Fig. 11). These data suggest a possible link between FADD/MKRN1 and survival during melanoma tumour progression.

### ADT-OH further enhances the apoptosis induced by FADD

**Fig. 3:**
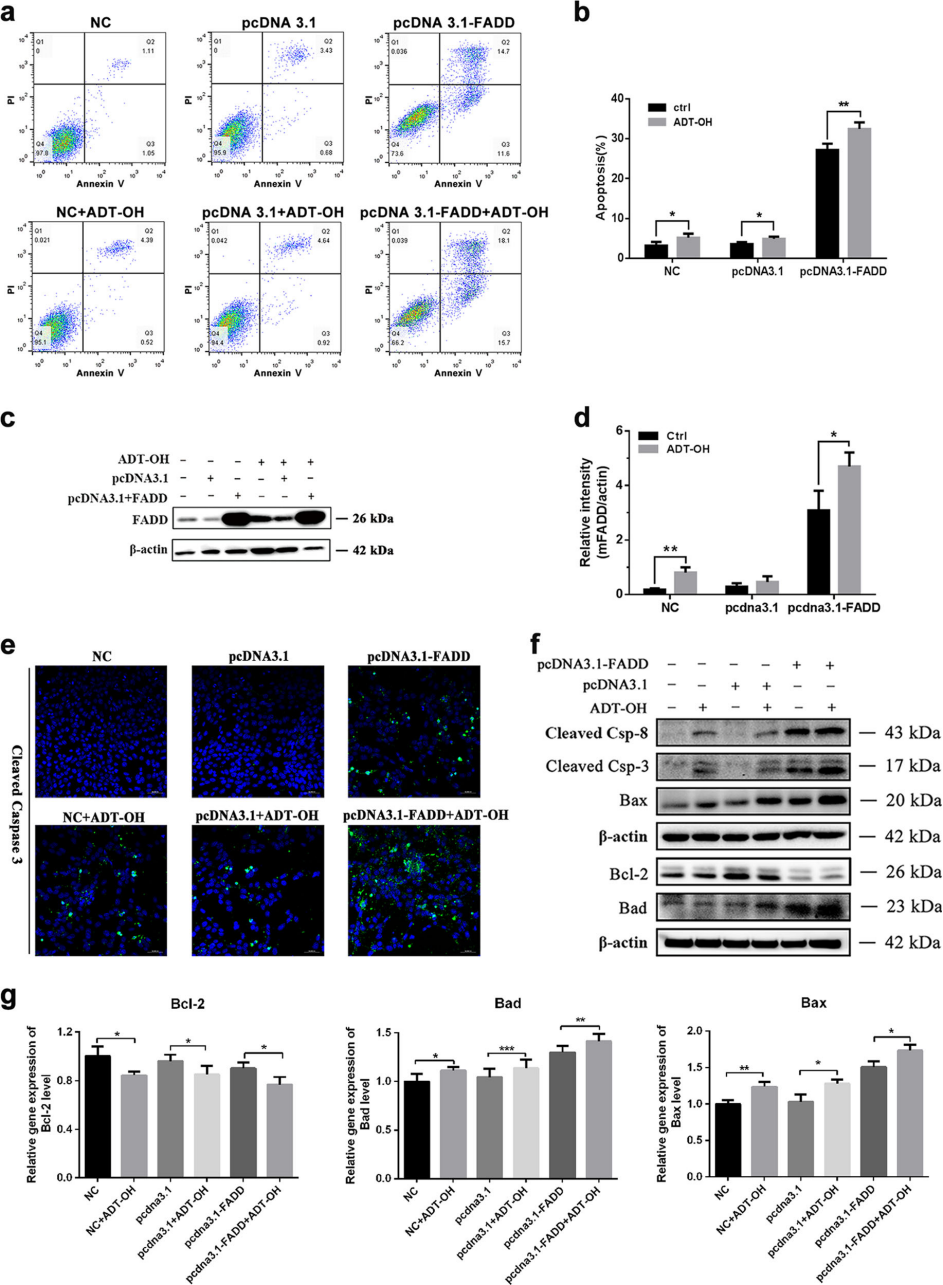
ADT-OH further enhances the apoptosis induced by FADD. **a** Flow cytometric analysis of B16F10 cells treated with ADT-OH (2 μM) after transfection of pcDNA 3.1-FADD or pcDNA 3.1 vectors for 16 h. **b** Quantitative analysis of apoptosis as detected in a (mean ± SD, *n* = 3 independent experiments). **c** Detection of FADD expression levels by WB analysis after ADT-OH (2 μM) stimulation or transfection of FADD or empty plasmid in B16F10 cells. **d** Band intensity shown in **c** was quantified by Image J software. **e** Representative immunofluorescence staining for cleaved caspase-3 (green) after ADT-OH treatment and transfection of FADD or empty vectors for 16 h. **f** Relative expression levels of Bcl-2, Bax, Bad, cleaved-caspase 8 and cleaved-caspase 3 after ADT-OH stimulation or transfection of FADD or empty plasmid in B16F10 cells were detected by WB analysis using indicated antibodies. **g** Relative mRNA levels of Bcl-2, Bad and Bax after ADT-OH stimulation or transfection of FADD or empty plasmid in B16F10 cells were detected by qPCR analysis. *P < 0.05, **P < 0.01, compared with the control group.

FADD overexpression can initiate apoptosis through self-association, through which it forms large filamentous aggregates called death effect filaments^33^. Here, we determined whether overexpression of mouse FADD or the combination of mouse FADD overexpression with ADT-OH treatment in B16F10 melanoma cells induces more apoptosis. As shown in Fig. 3a, b, compared with empty pcDNA3.1 vector transfection, overexpression of FADD significantly increased the number of apoptotic B16F10 cells. Transfection of pcDNA3.1-FADD led to death in approximately 27% of the cells, while pcDNA3.1 transfection led to death in 5% of the cells (Fig. 3a, b). When FADD was overexpressed in combination with ADT-OH treatment, the apoptotic ratio was further increased to 44%. In addition, the amount of FADD expression also changed following the same trend (Fig. 3c, d).

Siegel et al.^33^ previously reported that the FADD protein has the potential to form protein aggregates, which serve as platforms for pro-caspase-8 recruitment and activation, resulting in the activation of caspase-3. Immunofluorescence staining of cleaved caspase-3 was performed and revealed that transfection with FADD alone or in combination with ADT-OH treatment induced the apoptosis of B16F10 cells. As shown in Fig. 3e, FADD overexpression alone activated caspase-3, and its combination with ADT-OH further enhanced the activation of caspase-3, which was confirmed by the results from Western blot analysis. In addition, transfection with pcDNA-FADD alone caused minimal elevation of Bax and Bad, while the combination treatment of ADT-OH with pcDNA-FADD transfection obviously increased the expression of these proteins and reduced Bcl-2 levels (Fig. 3f). All of these results suggest that 10 μM ADT-OH treatment induced the apoptosis of B16F10 cells through both intrinsic and extrinsic apoptosis pathways. This phenomenon was more pronounced when ADT-OH treatment was combined with FADD overexpression. However, the pattern of changes in the mRNA levels of Bax, Bad and Bcl-2 was similar to that in their protein levels (Fig. 3g). Taken together, the data show that the overexpression of FADD can increase the antitumour efficacy of ADT-OH, even at a low dose.

**Fig. 4:**
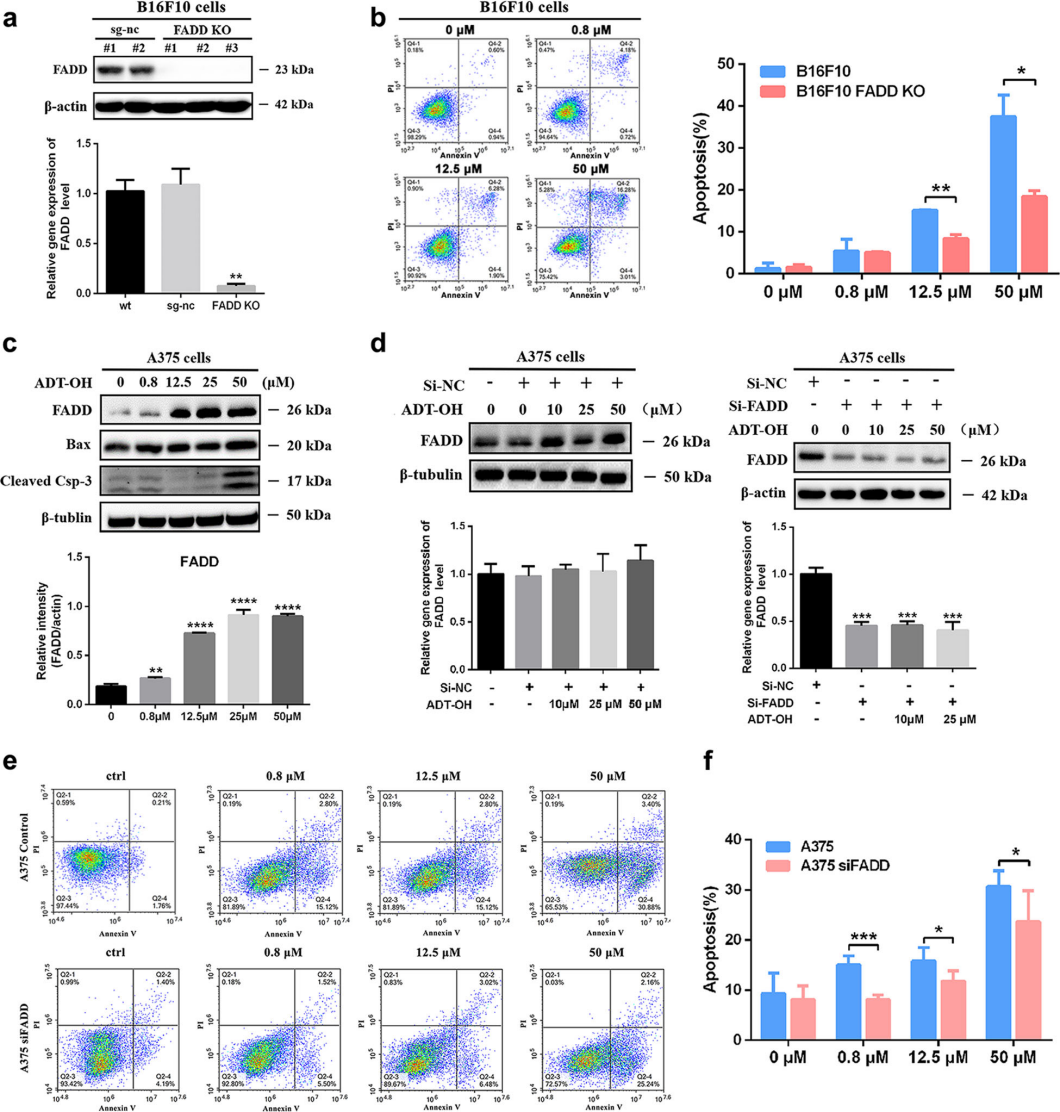
FADD is essential for ADT-OH-induced apoptosis. **a** Western blot analysis of FADD expression and mRNA levels of FADD in B16F10 transfection sg-nc and FADD KO cells after ADT-OH treatment. **b** B16F10 FADD KO cells were treated with ADT-OH at different concentration (0.8–50 μM) and apoptosis was determined by flow cytometry analysis (left). Quantitative analysis of apoptosis at various concentrations of exposure to ADT-OH (right). **c** Western blot analysis of FADD, Bad and Cleaved-Caspase 3 expression in A375 cells after ADT-OH treated in dose-dependent manner. **d** Detection of FADD expression levels by WB and qPCR analysis of FADD in A375 transfection si-NC and si-FADD interference fragment cells after ADT-OH treatment. **e**, **f** A375 control and interference FADD cells were treated with ADT-OH at different concentration (0.8–50 μM) and apoptosis was determined by flow cytometry analysis (**e**). Quantitative analysis of apoptosis at various concentrations of exposure to ADT-OH. Experiments (*n* = 3) were performed in triplicate (**c**, **e**). *P < 0.05, **P < 0.01, ****P < 0.001 compared with the vehicle group.

To further confirm whether the pro-apoptotic effect of ADT-OH was mediated by FADD, a FADD-knockout cell line was constructed with B16F10 cells (Fig. 4a). As the results show, the pro-apoptotic effect of ADT-OH was greatly blunted after FADD knockout (Fig. 4b). The apoptosis rate of FADD-KO cells after treatment with 50 μM ADT-OH was 22.26%, whereas that of wild-type B16F10 cells was 41.95%. Moreover, the same phenomenon was also found in the FADD-knockdown A375 and A549 cell lines (Fig. 4c–f, Supplementary Fig. 12). Consequently, we conclude that FADD might play an important role in ADT-OH-induced apoptosis.

### ADT-OH inhibits the growth of melanoma in vivo

**Fig. 5:**
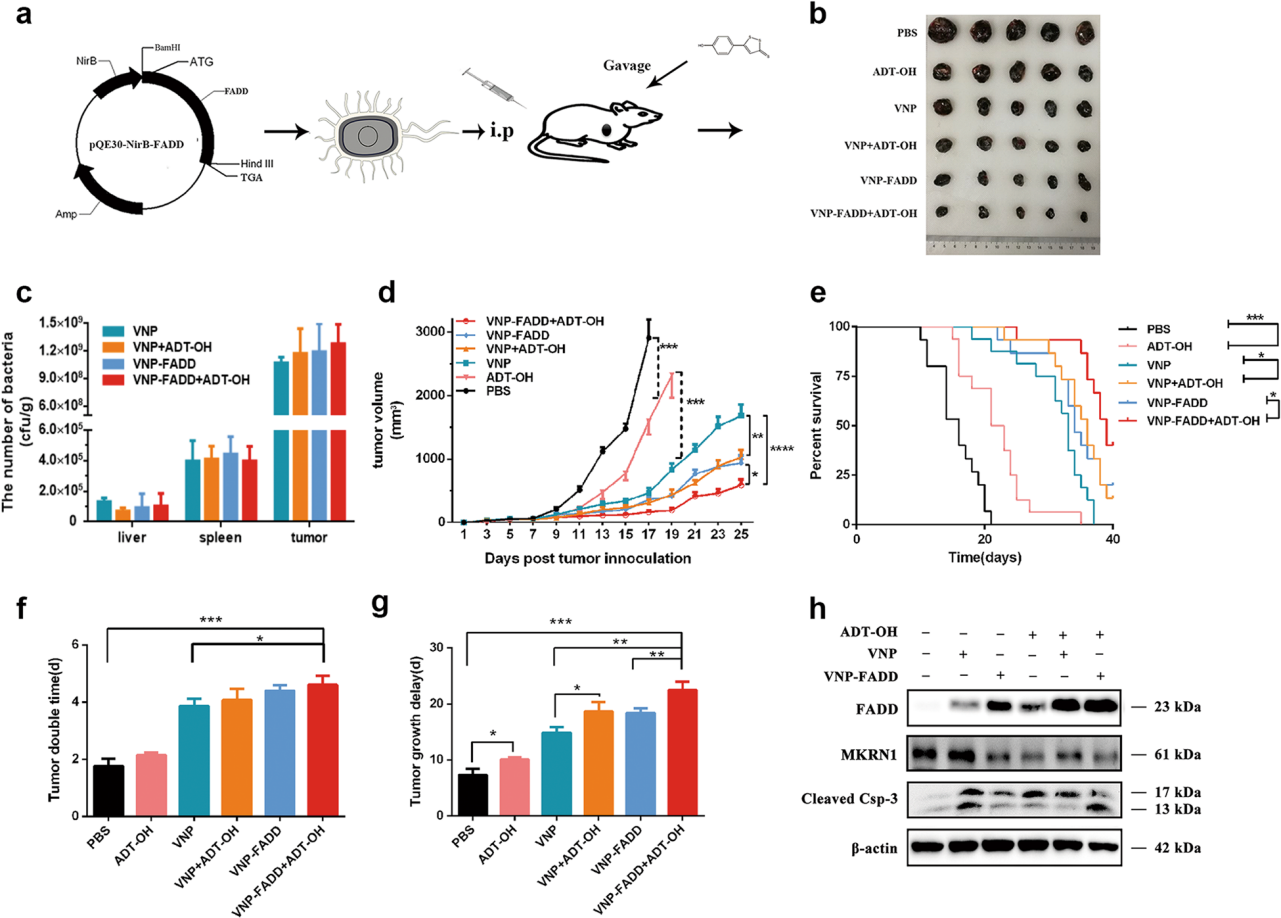
ADT-OH in combination with VNP-FADD significantly inhibits melanoma xenograft growth in vivo. **a** Animal treatment methods. **b** Representative therapeutic efficacy of ADT-OH combining with VNP strains for melanoma therapy. **c** The relative tissue distribution of the indicated stable strains was determined by colony formation assay. **d** Tumour growth curves. B16F10 tumour mice per group (*n* = 10 mice) were injected i.p. and gavage with PBS, ADT-OH, VNP, ADT-OH + VNP, VNP-FADD and ADT-OH + VNP-FADD. Tumour volumes among different groups were compared. Data are presented as mean ± SEM. *P < 0.05, **P < 0.01, ***P < 0.001. **e** Kaplan–Meier survival curves of mice bearing B16F10 melanomas receiving different treatments as indicated. Data were analysed by the log-rank test. *P < 0.05, **P < 0.01. Detailed statistics are attached to Supplementary Fig. 13. **f**, **g** Tumour doubling time and growth delay for each group. Data are presented as mean ± SD. *P < 0.05, **P < 0.01. **h** Detection of the expression of FADD, MKRN1 and cleaved-caspase 3 in tumour tissues of the melanoma bearing mice receiving different treatments by western blotting analysis. β-actin was served as loading control and band intensity quantified by Image J software is also shown in the right. *P < 0.05, **P < 0.01.

Together with the above in vitro results, ADT-OH administration combined with overexpression of FADD seems to be a promising strategy for melanoma treatment. *S. typhimurium* is a facultative anaerobic gram-negative bacterium and one of the most popular tumour therapy strains because of its tumour targeting ability and genetic stability^34,35^. Currently, an increasing number of studies have used VNP as a tumour drug delivery vehicle for the purpose of highly safe and highly efficient delivery^16,36^. A recombinant Salmonella strain for tumour-specific delivery of FADD, VNP-FADD, was successfully constructed in our laboratory previously^16^ (Fig. 5a), although it lacks efficient antitumour effects and fails to prolong survival in tumour-bearing mice. Therefore, this study aimed to combine the sustained H_2_S-release donor ADT-OH with VNP-FADD to enhance the antitumour effect of FADD. On one hand, the stability of FADD in tumour cells would be increased by ADT-OH, and on the other hand, VNP-FADD would deliver FADD specifically to the tumour; thus, the antitumour effect would be more comprehensive and effective than either intervention alone. To investigate the role of ADT-OH and FADD in the treatment of melanoma in vivo, a murine model of melanoma was generated as described above (Fig. 5a). First, we determined the biological distribution of the bacteria. The mice bearing B16F10 melanoma xenografts were injected with VNP and VNP-FADD and given ADT-OH by gavage at the same time. Three days later, the mice were sacrificed, and the tumour tissues were homogenised to analyse the bacterial titre. Colony formation experiments showed that ADT-OH treatment did not change the tumour target distribution of VNP (Fig. 5c).

**Fig. 6:**
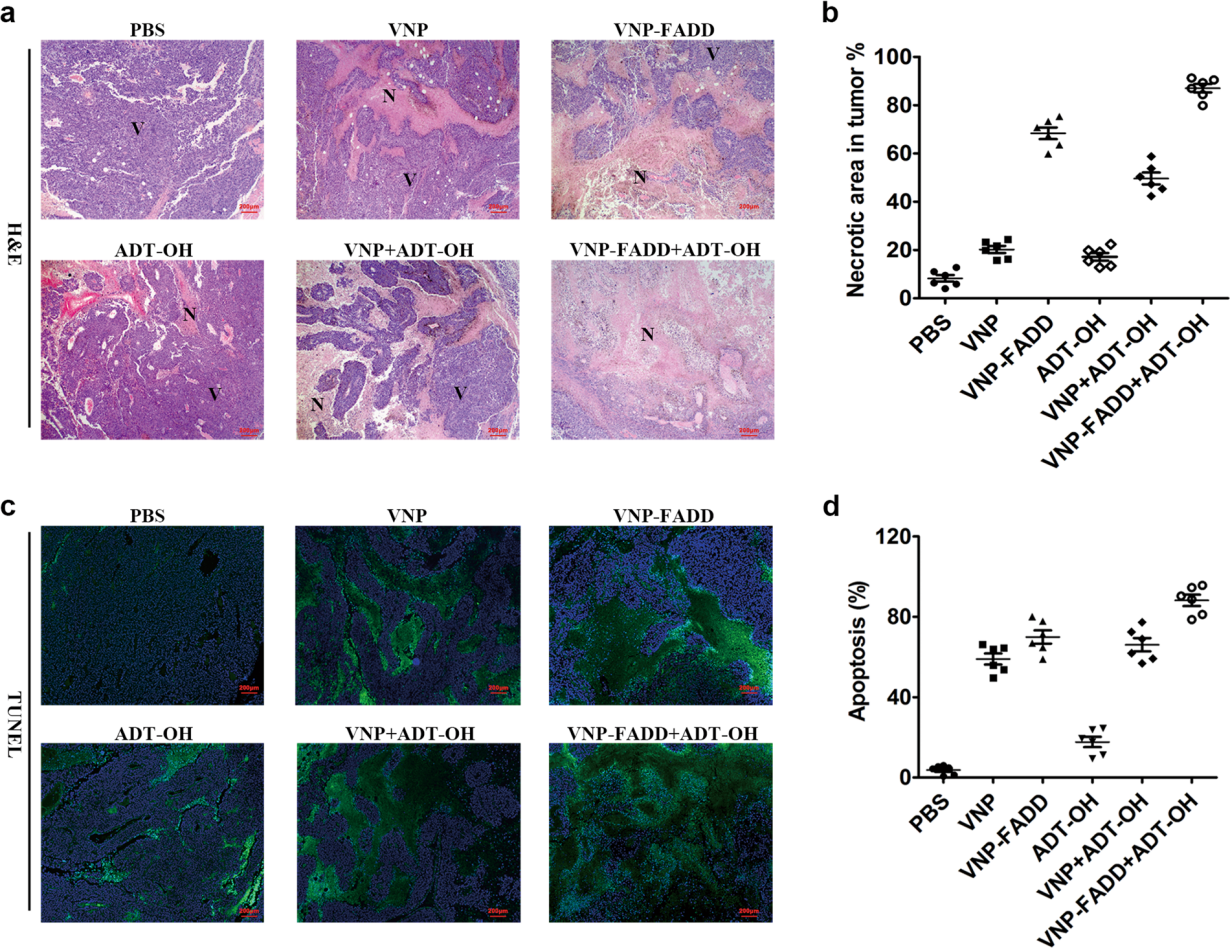
ADT-OH combine with VNP-FADD significantly induces necrosis of tumour and promotes melanoma cell apoptosis. **a** H&E staining of the tumour sections. The representative images (×50) showed necrotic areas of B16F10 tumour tissue treated with PBS, ADT-OH, VNP, ADT-OH + VNP, VNP-FADD and ADT-OH + VNP-FADD; N necrotic tumour regions, V vital tumour regions. **b** Quota for tumour necrosis. Software Image J was used to determine tumour necrosis. Two sections/mouse and three mice were prepared (mean ± SD, *P *<* 0.05, **P < 0.01). **c** TUNEL assay was used to detect apoptotic B16F10 melanoma cells from the mice treated with PBS, ADT-OH, VNP, ADT-OH+VNP, VNP-FADD and ADT-OH + VNP-FADD. **d** Proportion of apoptotic cells to total cells: TUNEL-positive cells were counted from three fields with the highest positive staining cell density in each section to determine the percentage of apoptotic cells (mean ± SD, *P < 0.05, **P < 0.01).

The tumour suppressing potency of ADT-OH combined with VNP-FADD was evaluated using a B16F10 mouse model. The xenograft mice were randomly divided into six groups and treated with PBS, ADT-OH, VNP, VNP+ADT-OH, VNP-FADD and VNP-FADD+ADT-OH. As shown in Fig. 5b, d, the tumour volume in the group of mice that received ADT-OH treatment was significantly smaller than that of the non-ADT-OH treatment groups. Owing to the certain antitumour effect of VNP, mice treated with VNP had a smaller average tumour volume than the control mice in this study. However, the tumour volume of the VNP-FADD groups was significantly smaller than that of the VNP groups. The VNP-FADD+ADT-OH group had the most prominent outcome, as the final tumour volume was the smallest in the VNP-FADD+ADT-OH group among all the groups (*P* < 0.001). Furthermore, the survival time of the tumour-bearing mice after ADT-OH and VNP-FADD treatment was prolonged significantly (Fig. 5e, Supplementary Fig. 13). In Fig. 5f, the tumour doubling time of the VNP-FADD+ADT-OH group was prolonged significantly compared with that of the other groups. In addition, the groups receiving the combined ADT-OH treatment had significantly increased tumour doubling time and tumour growth delay compared with the non-ADT-OH treatment groups (*P* < 0.05) (Fig. 5g). These results showed that the VNP-FADD+ADT-OH group exhibited the longest tumour growth delay: the tumour doubling time was 13.65 d (confidence interval (CI): 12.91–14.48 d) in the PBS group, 16.12 d (CI: 15.56–16.72 d) in the ADT-OH group, 20.86 d (CI: 19.85–21.99 d) in the VNP group, 24.32 d (CI: 23.72–24.96 d) in the VNP + ADT-OH group, 24.06 d (CI: 22.87–25.38 d) in the VNP-FADD group and 29.63 d (CI: 27.38–32.28 d) in the VNP-FADD + ADT-OH group. Western blot analysis showed that FADD was overexpressed in the tumours after VNP-FADD treatment; furthermore, the protein levels of FADD and cleaved caspase-3 were upregulated after the combined ADT-OH treatment (Fig. 5h). Immunofluorescence staining with the anti-FADD (N-term) antibody confirmed this result (Supplementary Fig. 14). As expected, FADD preferentially accumulated in the necrotic area of the tumour, indicating that FADD was successfully and highly expressed in the tumour hypoxic region. Moreover, the H&E staining and TUNEL assays showed that all groups except the PBS group had notable tumour necrosis areas, while the groups that received the ADT-OH combination treatment much greater necrosis areas than did the non-ADT-OH treatment groups (Fig. 6a, b). Notably, the VNP-FADD + ADT-OH group had the most severe cell death, and mice from this group developed the largest necrotic areas among those in all the treatment groups. In addition, the in situ TUNEL assay of the tumour sections showed the same trend. More TUNEL-positive cells were detected in the VNP-FADD + ADT-OH group, suggesting that the combination strategy induced higher levels of apoptosis (Fig. 6c, d). Briefly, these results indicated that treatment with ADT-OH in combination with VNP-FADD significantly increased the protein level of FADD in tumour tissues and then induced apoptosis, thereby inhibiting tumour growth and improving the survival rate of tumour-bearing mice.

### FADD knockout greatly blunts the antitumour effect of ADT-OH in vivo

**Fig. 7:**
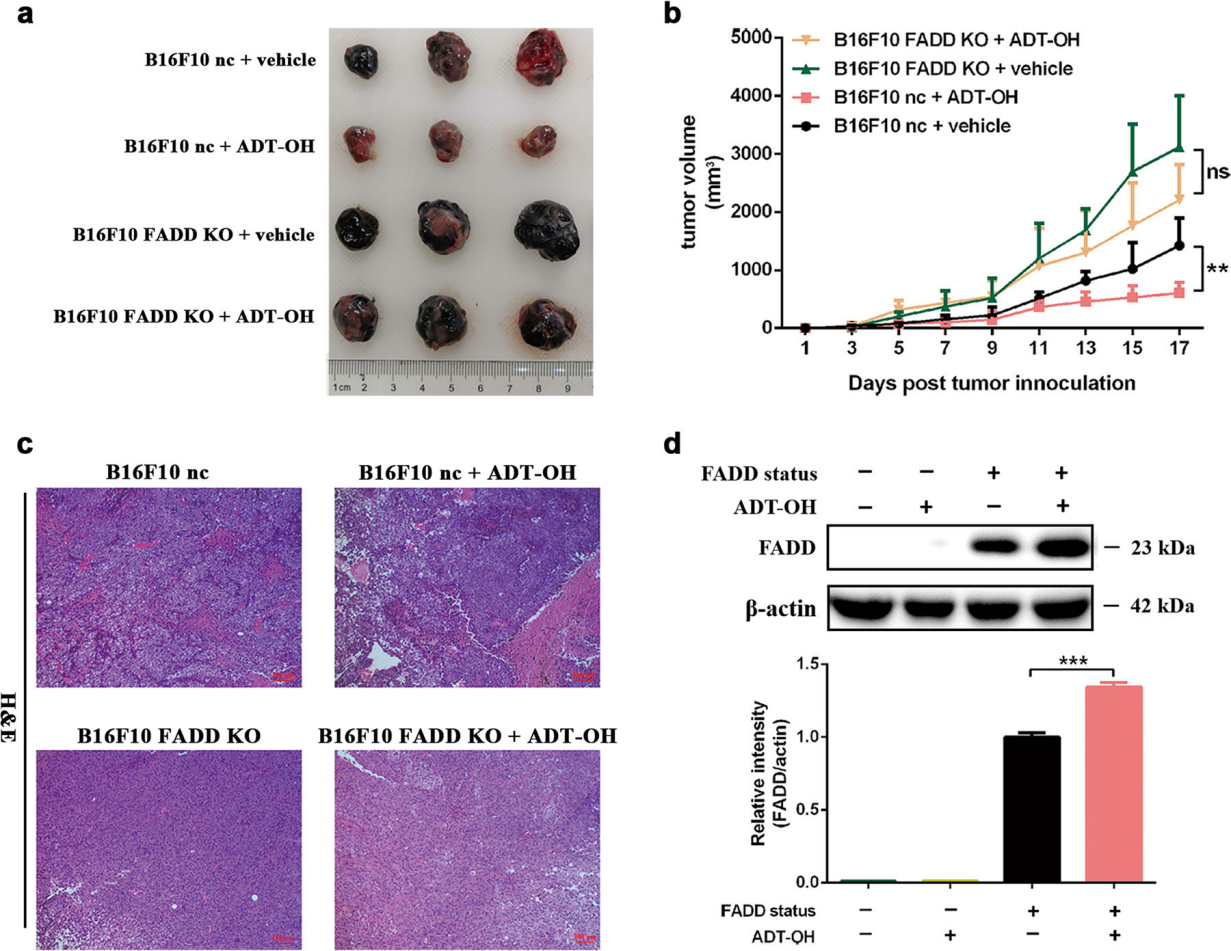
ADT-OH inhibits melanoma xenograft growth in vivo but has little effect on FADD knockout melanoma. **a** Representative therapeutic efficacy of ADT-OH for B16F10 nc and B16F10 FADD KO melanoma therapy. **b** Tumour growth curves. B16F10 nc and B16F10 FADD KO tumour mice per group (*n* = 8 mice) were gavage with vehicle, ADT-OH. Tumour volumes among different groups were compared. Data are presented as mean ± SEM. **P < 0.01. **c** H&E staining of the tumour sections. The representative images (100×) showed necrotic areas of B16F10 nc and B16F10 FADD KO tumour tissue treated with vehicle and ADT-OH; N necrotic tumour regions, V vital tumour regions. **d** Western blots for FADD expression by B16F10 FADD KO vs B16F10 nc tumour tissues after vehicle and ADT-OH (37.5 mg/kg) treatment. Data are presented as mean ± SD. ***P < 0.001.

Next, to further demonstrate the role of FADD in the treatment of melanoma in vivo with ADT-OH, we constructed a mouse melanoma model by subcutaneously injecting B16F10 cells or B16F10 FADD-KO cells into C57BL/6 mice. The melanomas derived from the FADD-knockout cells stably grew to 3000 mm^3^ within 14 d, and there was no significant change in tumour size after ADT-OH treatment. In contrast, the tumours derived from the normal melanoma cells grew to only 1400 mm^3^; however, ADT-OH treatment reduced the tumour size to 600 mm^3^ (Fig. 7a, b). In Fig. 7c, the H&E staining assays also showed the same trend as that in Fig. 7b. ADT-OH treatment significantly increased the tumour necrosis area of B16F10 nc cells, but these effects were eliminated after FADD knockout. In addition, as shown in Fig. 7d, FADD was successfully knocked out, and this cell line responded minimally to ADT-OH treatment in vivo. Taken together, the data from our study suggest that FADD plays a critical role in the pro-apoptotic and antitumour effects of ADT-OH and that the absence of FADD greatly blunts these effects.

## Discussion

Melanoma remains the deadliest and most aggressive form of skin cancer. Conventional therapy does not effectively inhibit tumour relapse and often results in therapy resistance, often within a few months^9,37^. In many cases, the apoptosis of tumour cells has been suppressed, resulting in cancer cell survival and hyperproliferation followed by resistance to chemotherapy. Thus, the enhancement of apoptosis is considered to be an attractive and effective strategy for cancer prevention and treatment. Apoptosis resistance often occurs after treatment with chemotherapy drugs such as TMZ and DTIC, which results in poor clinical efficacy. Therefore, the development of more effective and safe pro-apoptotic drugs is crucial to the treatment of melanoma.

Accumulating studies have shown that H_2_S plays an important role in cancer biological processes, although H_2_S has opposing effects: pro-cancer and anticancer. A recent study found that ROS levels in thyroid cancer cells decreased after 24 h of treatment with 20–50 μM NaHS, which activated the PI3K/AKT/mTOR signalling pathway to promote tumour cell proliferation and migration^25^. However, some other H_2_S donors were reported to inhibit tumour cell proliferation and viability significantly. For example, GYY4137 (200–400 μM) treatment for 24 h triggered cell cycle arrest through the downregulation of cyclin D1 in HepG2 cells in a dose-dependent manner^28^. Treatment with H_2_S gas (1.1 μM) for 48–72 h significantly induced apoptosis of CA9-22 cancer cells, whereas there was no pro-apoptotic effect on normal human gingival keratinocytes^38^. Therefore, it should be noted that higher concentrations of exogenous H_2_S or exposure to lower levels of H_2_S over a relatively long period of time could selectively inhibit cancer cell proliferation and induce apoptosis. Thus, H_2_S sustained-release donors are potential anticancer agents.

Recently, several H_2_S donors and H_2_S-releasing hybrids, namely, DATS, ATB-346 and GYY4137, have been developed and designed as novel anticancer drugs. For example, GYY4137 induced the apoptosis of various types of cancer cells, including HeLa, HCT-116, Hep G2, HL-60, MCF-7, MV4-11 and U2OS cells, with obvious inhibitory effects on tumour growth; however, limited application has been undertaken in melanoma treatment^39^. On the other hand, the slow-releasing H_2_S donor 5-(4-hydroxyphenyl)-3H-1,2-dithiole-3-thione (ADT-OH) is known as a potent therapeutic with chemopreventive and cytoprotective properties due to its 3*H*-1,2-dithiole-3-thione (dithiolethione) group, which is also one of the most widely used moieties for synthesising slow-releasing organic H_2_S donors^39,40^. Here, we further broadened the potential antitumour effect of ADT-OH by demonstrating its ability to inhibit melanoma progression in vitro and in vivo. Interestingly, many studies have shown that H_2_S has little toxic effect on normal cells but can significantly inhibit tumour cells^25,38,41^. In vitro, our results showed that ADT-OH induced more apoptosis in tumour cells than in normal cells, indicating that ADT-OH has potential in the treatment of melanoma with little side effects. Additionally, our study demonstrated that ADT-OH significantly increased the apoptosis of B16F10 cells by enhancing the activation of caspase-3. Previous reports showed that H_2_S donors inhibit the activity of NF-κB and decrease the expression of anti-apoptotic proteins such as Bcl-2 to induce spontaneous apoptosis of human melanoma cells^26^. Interestingly, in this study, we found that ADT-OH could not only induce apoptosis through this reported intrinsic apoptotic pathway but also enhance the extrinsic apoptotic pathway, as evidenced by the cleavage of caspase-8. As a classical apoptosis adaptor protein, FADD is indispensable for the induction of extrinsic apoptosis. Here, our study showed that ADT-OH significantly increased the protein level of FADD in several cancer cell lines, including B16F10 melanoma cells. Furthermore, our results demonstrated that the pro-apoptotic effect of ADT-OH treatment was mainly mediated through the inhibition of ubiquitin-induced degradation of FADD. In addition, our results showed that ADT-OH significantly decreased the protein stability of MKRN1. A previous study reported that upregulation of MKRN1 resulted in ubiquitin-induced degradation of FADD in conjunction with tumorigenesis of breast cancer^32^. Interestingly, our results suggested that ADT-OH might increase the stability of FADD by decreasing the stability of MKRN1. In addition, our results showed that FADD overexpression combined with ADT-OH treatment more strongly induced apoptosis though the enhancement of both the intrinsic and extrinsic apoptotic pathways than either treatment alone in vitro. In dissecting the role of FADD in the treatment of melanoma with ADT-OH, we performed additional experiments using FADD-knockout B16F10 cell lines. The data showed that the FADD-KO melanoma cells had little response to ADT-OH and that the level of apoptosis induced by ADT-OH was much lower than that of the wild-type cells. In addition, in vivo experiments showed that ADT-OH had no significant therapeutic effect on FADD-knockout melanoma cells. Altogether, these results show that FADD knockout greatly blunts the pro-apoptotic effect of ADT-OH and indicate that FADD plays an important role in the treatment of melanoma with ADT-OH.

Since ADT-OH treatment combined with overexpression of FADD induced apparent apoptosis of melanoma cells in vitro, we further explored their combined effects on melanoma cell apoptosis in vivo. Our team previously showed that FADD could be delivered into tumour cells successfully by attenuated *S. typhimurium* VNP20009 under the control of the NirB promoter (VNP-FADD)-targeted delivery^16^. In this current study, VNP-FADD induced the overexpression of FADD in tumour tissues, which was further increased after ADT-OH treatment. In vivo experiments showed that treatment with ADT-OH reduced tumour volume and weight. Intriguingly, VNP-FADD combined with ADT-OH treatment exhibited greater antitumour efficacy than VNP-FADD or ADT-OH treatment alone, as indicated by the marked inhibition of tumour growth and the prolonged survival of tumour-bearing mice. In addition, it should be noted that the dose of ADT-OH used in our study was less than one-half the dose of other H_2_S donors reportedly used in previous studies^26,39^. In addition, in this study, the amounts of VNP and VNP-FADD at 1 × 10^5^ cfu per mouse were also lower than those used in previous studies^16,42^. All of these results indicate that FADD overexpression combined with ADT-OH treatment exhibits excellent tumour suppression effects with little side effects.

**Fig. 8:**
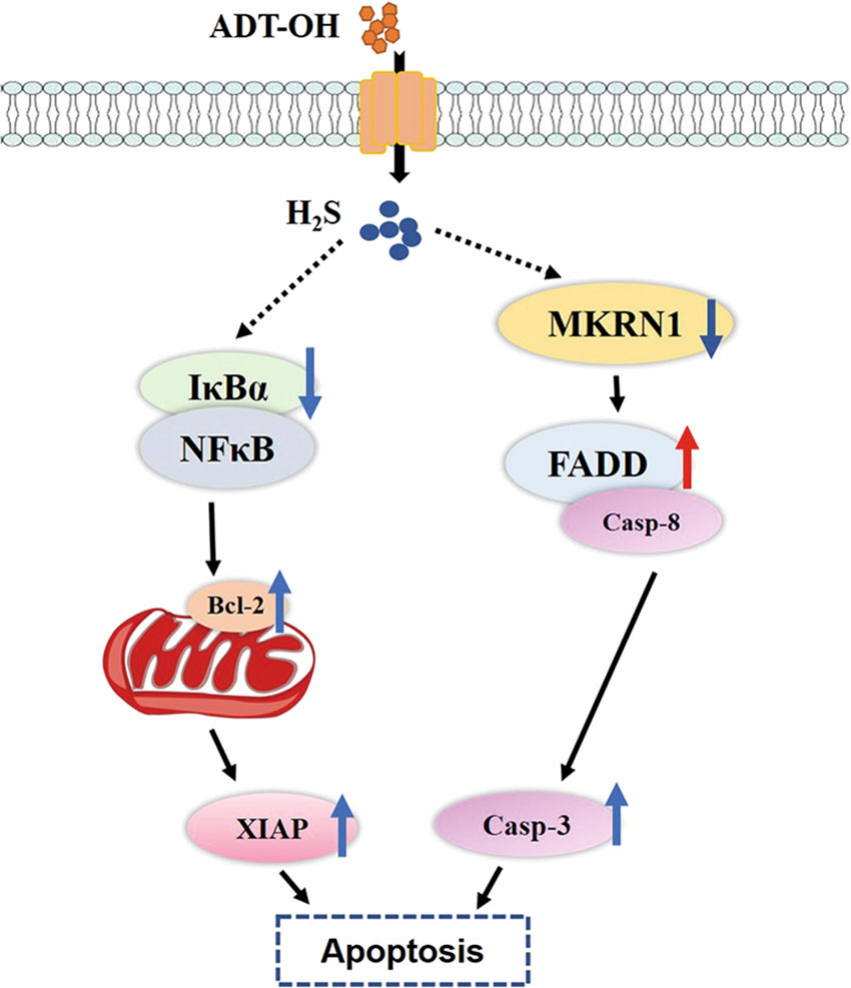
A proposed signalling pathway by which ADT-OH induces cell death in B16F10 cells. ADT-OH leads to apoptosis of B16F10 cells via FADD-dependent apoptotic pathway. In B16F10 cells, ADT-OH promotes IκBα degradation and inhibits NF-κB activation, thus leading to down-regulate NF-κB-targeted anti-apoptotic genes, such as XIAP, BCL2, to finally induce apoptosis. In addition, ADT-OH can also increase the expression level of intracellular FADD by decreasing the expression level of MKRN1, an E3 ubiquitin ligase of FADD, thereby activating caspase 8 in downstream to further induce caspase 3 activation, finally enhancing apoptosis.

In summary, our study shows, for the first time, that the slow-releasing H_2_S donor ADT-OH is a potential antitumour agent because it enhances the FADD-dependent extrinsic apoptosis of melanoma cells (Fig. 8). We also demonstrate that the combination of ADT-OH with VNP-FADD may provide a promising alternative therapy for melanoma, although further studies are needed to evaluate its potential in clinical applications.

## Supplementary information

Supplemenatal Legends

Supplementary tables

Supplementary Figure 1

Supplementary Figure 2

Supplementary Figure 3

Supplementary Figure 4

Supplementary Figure 5

Supplementary Figure 6

Supplementary Figure 7

Supplementary Figure 8

Supplementary Figure 9

Supplementary Figure 10

Supplementary Figure 11

Supplementary Figure 12

Supplementary Figure 13

Supplementary Figure 14
