# Supplementary material for: ADT-OH, a hydrogen sulfide-releasing donor, induces apoptosis and inhibits the development of melanoma in vivo by upregulating FADD
Source: Cell Death Dis. 2020 Jan 16;11(1):33. doi: 10.1038/s41419-020-2222-9 (PMC6965651; doi:10.1038/s41419-020-2222-9)
Supplement: Supplementary file 1 — Supplemenatal Legends [file 41419_2020_2222_MOESM1_ESM.docx]

**Supplementary Tables**

**Table 1:** **Target sequences of sgRNA against mouse FADD.**

**Table 2:** **The primers of selected genes for real time PCR.**

**Supplementary Figures**

**Supplementary Figure S1: ADT-OH, the slow-releasing organic hydrogen sulfide donor.** H_2_S measurement released by B16F10 melanoma cells after ADT-OH treatment at different time point as indicated. Data are represented as mean ± SD for different experiments performed in duplicate. **P* < 0.05, ***P* < 0.01 compared with vehicle group.

**Supplementary Figure S2:** **ADT-OH inhibits tumor cell proliferation.** A549, H446, H1299, HepG2, F1(B16F1), A375, HCT-116 and MDA231(MDA-MB-231) cells were incubated with increasing concentrations of ADT-OH for 24 h. Cell viability was determined by CCK-8 assay. **P* < 0.05, ***P* < 0.01,****P* < 0.005, *****P* < 0.001, compared with the vehicle group.

**Supplementary Figure S3: ADT-OH induces tumor cell apoptosis but has a slight effect on normal cells.** (A) HaCaT (human immortalized epidermal cells) and HK2 cells were treated with ADT-OH at different concentration (0.8-50 μM) and apoptosis was determined by flow cytometry analysis. And quantitative analysis of apoptosis at various concentrations of exposure to ADT-OH. Experiments (n = 3) were performed in triplicate. (B) Representative FACS analysis and quantitative analysis of Annexin V and propidium iodide (PI) staining after ADT-OH treated at different concentration (0.8-50 μM) in HepG2, MCF-7, HCT-116 and MDA-MB-231 cells. Data are represented as mean ± SD for different experiments performed in duplicate. **P* < 0.05, ***P* < 0.01, ****P* < 0.005, *****P* < 0.001, compared with the vehicle group.

**Supplementary Figure S4: ROS production.** The ROS concentration was determined after B16F10 cells treated with ADT-OH (2 μM, 10 μM and 50 μM) for 24 hours. Data are represented as mean ± SD for different experiments performed in duplicate. ****P* < 0.005 compared with the vehicle group.

**Supplementary Figure S5: ADT-OH increases extrinsic apoptosis by increasing FADD expression.** Statistical analysis of Figure 2a by Image J. Western blot analysis of FADD, XIAP, Bcl-2, cleaved caspase-8, cleaved caspase-3 and cleaved PARP in B16F10 cells after ADT-OH (10μM) treated in time-dependent manner. **P* < 0.05, ***P* < 0.01, ****P* < 0.005, *****P* < 0.001, compared with the vehicle group.

**Supplementary Figure S6: Incubation of B16F10 cells with ADT-OH (10 μM) resulted in inhibition of** **IκBα degradation.** Western blot analysis carried out on the cytosolic extracts obtained from B16F10 cells treated with ADT-OH (10 μM) for 15, 30, or 60 min shows an inhibition of IκBα degradation. ***P* < 0.01, *****P* < 0.001, compared with the vehicle group.

**Supplementary Figure S7: ADT-OH increases the expression of FADD in various tumor cells.** (A-D) Western blot analysis of FADD expression after ADT-OH stimulation in dose-dependent manner in 4T1 cells (A), LLC cells (B), A549 cells (C) and HepG2 cells (D). Statistical analysis of western blot by Image J below the respective Western blot plots; ****P* < 0.005, *****P* < 0.001, compared with the vehicle group.

**Supplementary Figure S8: ADT-OH suppresses the ubiquitin degradation of FADD.** Statistical analysis of Figure 2e by Image J.

**Supplementary Figure S9: ADT-OH reduces FADD ubiquitination by regulating MKRN1.** Statistical analysis of Figure 2g by Image J. Western blot analysis of FADD and MKRN1 in B16F10 cells after NaHS and ADT-OH (10μM) treated. **P* < 0.05, ***P* < 0.01, ****P* < 0.005, *****P* < 0.001, compared with the vehicle group.

**Supplementary Figure S10: ADT-OH reduces FADD ubiquitination by regulating MKRN1.** (A) The MKRN1 mRNA level of B16F10 cells were detected by qPCR analysis after ADT-OH treatment for 1 and 6 h respectively. (B) After transfection with MKRN1, B16F10 cells were treated with 40 μg/ml of CHX for the indicated time to determine protein stability of MKRN1 in the absence or presence of ADT-OH (10 μM). Cells were lysed and analyzed by WB using anti-FADD and anti-actin antibodies. **P* < 0.05, ***P* < 0.01. Data are expressed as mean ± SD of three independent experiments.

**Supplementary Figure S11: The level of the MKRN1 gene is associated with the prognosis of melanoma patients.** Kaplan-Meier curves for recurrence-free survival were created using a Kaplan-Meier plotter (www.kmplot.com), in which melanoma patients were classified according to high and low MKRN1 gene expression. The hazard ratio (with 95% confidence interval) and the log rank p value were calculated.

**Supplementary Figure S12: FADD is essential for ADT-OH-induced apoptosis.** (A) Western blot analysis of FADD, Bad and Cleaved-Caspase 3 expression in A549 cells after ADT-OH treated in dose-dependent manner. (B) Detection of FADD expression levels by WB and qPCR analysis of FADD in A549 transfection si-NC and si-FADD interference fragment cells after ADT-OH treatment. (C-D) A549 control and interference FADD cells were treated with ADT-OH at different concentration (0.8-50 μM) and apoptosis was determined by flow cytometry analysis (C). Quantitative analysis of apoptosis at various concentrations of exposure to ADT-OH (D). Experiments (n = 3) were performed in triplicate (C). ***P* < 0.01, ****P* < 0.005, *****P* < 0.001 compared with the vehicle group.

**Supplementary Figure S13: Detailed statistics of Fig. 6e.**

**Supplementary Figure S14: ADT-OH further enhances the apoptosis induced by FADD.** (A) Representative immunofluorescence staining for FADD (red) of B16F10 tumor tissue treated with PBS, ADT-OH, VNP, ADT-OH + VNP, VNP-FADD and ADT-OH + VNP-FADD. (B) Quota for the expression level of FADD in tumor tissue. Software Image J was used to determine. Two sections/mouse and three mice were prepared (mean ± SD, **P* < 0.05, ****P* < 0.05).
