# Supplementary material for: ADT-OH, a hydrogen sulfide-releasing donor, induces apoptosis and inhibits the development of melanoma in vivo by upregulating FADD
Source: Cell Death Dis. 2020 Jan 16;11(1):33. doi: 10.1038/s41419-020-2222-9 (PMC6965651; doi:10.1038/s41419-020-2222-9)
Supplement: Supplementary file 2 — Supplementary tables [file 41419_2020_2222_MOESM2_ESM.docx]

**ADT-OH, a hydrogen sulfide-releasing donor, induces apoptosis and inhibits development of melanoma *in vivo* by upregulation of FADD**

**Fangfang Cai^1^, Nini Cao^1^, Xiangyu Zhang^1^, Jia Liu^1^, Huangru Xu^1^, Yanan Lu^1^, Jia Chen^1^, Yunwen Yang^1^, Jian Cheng^2,*^, Zi-Chun Hua^1,3,*^, Hongqin Zhuang^1,*^**

**Table 1:** **Target sequences of sgRNA against mouse FADD.**

| sgRNA | Target sequences |
| --- | --- |
| FADD-1 | CACCGTACCCCCGAAGTCTGAGTGA |
| FADD-2 | CACCGTGCCCGACAGGCTGCCGGACA |
| negative control (nc) | CACCGCGCCAAACGTGCCCTGACGG |

**Table 2:** **The primers of selected genes for real time PCR.**

| Gene | Forward primer (5’-3’) | Reverse primer (5’-3’) |
| --- | --- | --- |
| Bad | TGAGCCGAGTGAGCAGGAA | GCCTCCATGATGACTGTTGGT |
| Bax | AGACAGGGGCCTTTTTGCTAC | AATTCGCCGGAGACACTCG |
| Bcl-2 | GCTACCGTCGTGACTTCGC | GCTACCGTCGTGACTTCGC |
| FADD | GCGCCGACACGATCTACTG | TTACCCGCTCACTCAGACTTC |
| MKRN1 | CTGAGTGCAAAACCATCCCTC | CTGAACCTGCTCCTACAGTTG |
| β-actin | GAGACCTTCAACACCCCAGC | ATGTCACGCACGATTT CCC |
